# In Commemoration of Dr. Mostafa Pourtaghva Shahrestani, a Pioneer in Infectious Disease Research

**DOI:** 10.34172/aim.2024.16

**Published:** 2024-02-01

**Authors:** Fatemeh Bardestani, Mohammad Ali Rad, Mohammad Hossein Azizi, Mohammad Mehdi Gouya, Ehsan Mostafavi

**Affiliations:** ^1^Research Centre for Emerging and Reemerging Infectious Diseases, Pasteur Institute of Iran, Tehran, Iran; ^2^Faculty of Veterinary Medicine, University of Tehran, Tehran, Iran; ^3^Academy of Medical Sciences, Tehran, Iran; ^4^School of Medicine, Iran University of Medical Sciences, Tehran, Iran

**Keywords:** Cholera, Plague, Smallpox, Tuberculosis

## Abstract

It is important to honor the contributions of scientific leaders who have dedicated their lives to advancing knowledge and serving their country. One way is to document their experiences and personalities in a documentary format, which can serve as a historical record and an inspiration for future generations. Dr. Mostafa Pourtaghva Shahrestani, a renowned physician and specialist in infectious diseases and tropical medicine, has made significant contributions to public health in Iran. He has played a crucial role in controlling infectious diseases such as smallpox, tuberculosis, rabies, plague, and cholera. Throughout his career, he has held various executive positions, including the head of Pasteur Hospital and the director of the Pasteur Institute of Iran. Dr. Pourtaghva’s life is a testament to his unwavering dedication to public health services, as evidenced by his continuous effort, love, and interest in honest work. His inspiring story can serve as a model for those who seek to follow in his footsteps.

## Childhood

 Mostafa Pourtaghva Shahrestani was born on December 1st, 1936, in the “Shahrestan” village in Rasht, Gilan Province, Iran. His childhood was marked by the devastating effects of the Second World War on Iran, which lasted from 1939 to 1945. In 1941, Britain and the Soviet Union invaded Iran, claiming the presence of Germans in the country. The occupation led to the destruction of infrastructure, food shortages, famine, and the spread of infectious diseases, resulting in a high mortality rate.^1^ In September 1941, Rasht was bombed and occupied by Russian forces, causing many residents to flee to safer regions.^2^ When Mostafa was approximately five years old, his family temporarily left Rasht and eventually settled in Tehran.

## Education

**Figure 1:**
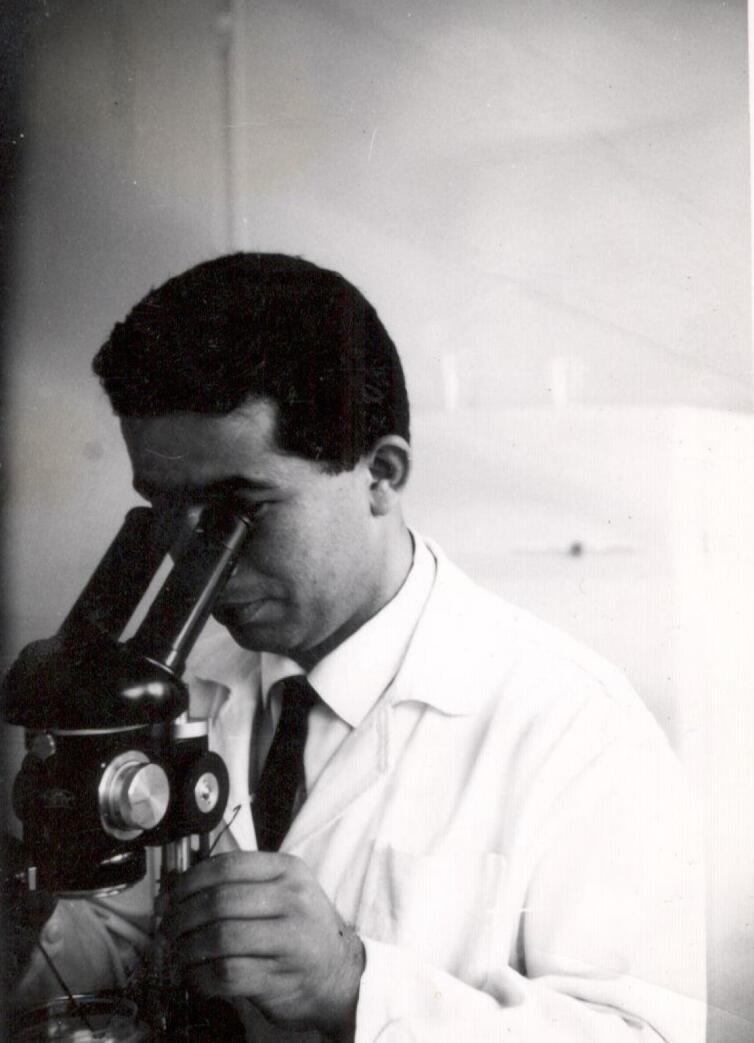
Dr. Mostafa Pourtaghva During His Medical Studies at the University of Tehran in 1968

**Figure 2:**
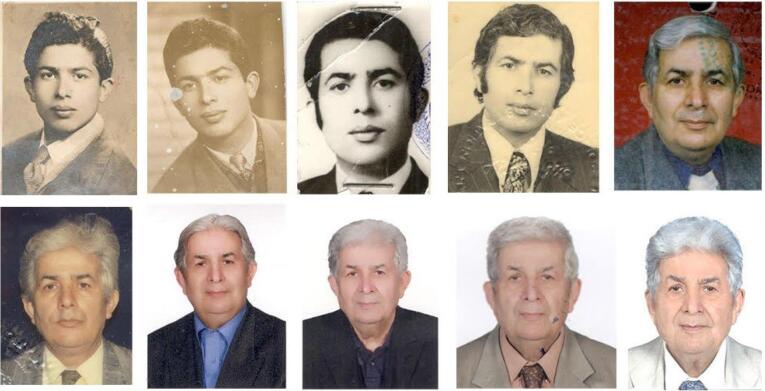
Dr. Mostafa Pourtaghva Over Time

 After completing his primary education in Rasht, he enrolled in the Pasteur Institute of Iran in 1955 and expedited his studies, finishing the remaining three years of high school within a year and obtaining his high school diploma in Tehran in 1956. In 1960, Mr. Pourtaghva was encouraged and supported by the director of the Pasteur Institute of Iran, Dr. Marcel Baltazard, to go to France and complete the pre-medical course at Montpellier University. Upon returning to Iran, he pursued his studies and eventually obtained a medical degree from the University of Tehran in 1968 (Figure 1). His doctorate in general medicine was entitled “Investigation of cases of botulism in Iran”.^3^ Dr. Pourtaghva also earned a master’s degree in tropical medicine from the University of Paris and the Pasteur Institute of Senegal in 1975. Additionally, he received a diploma in laboratory-diagnostic sciences from the University of Paris in 1976 and completed a virology course at the Pasteur Institute of Senegal in 1977 (Figure 2).

## Activities

**Figure 3:**
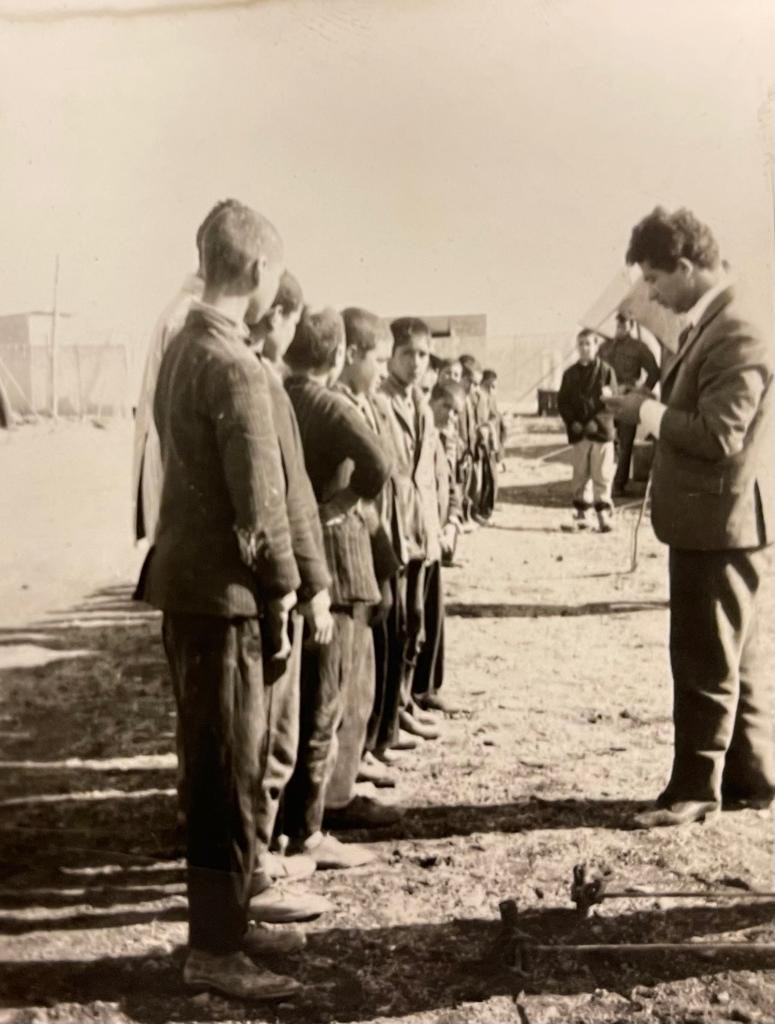
Providing Aid to Earthquake Victims in South Khorasan (Dasht-Biaz, Kakhak, and Ferdous) in September 1347. *Note*. Dr. Mostafa Pourtaghva, the head of the field hospital in Kakhak, which was established by the Germans, is examining affected individuals. Most people were suffering from ringworm

 Dr. Pourtaghva began his service at the Pasteur Institute of Iran in April 1956 as a daily wage worker. He started working at Pasteur as a third-class waiter (1956‒1959) and then continued his medical education. During his time at the Pasteur Institute of Iran, he held various positions, such as the head of the smallpox vaccine production laboratory (1965‒1971), the head of the tuberculosis department (1971‒1972),^4^ and the head of the Pasteur Hospital (1972‒1981). He also served in departments such as epidemiology, B.C.G. vaccine production, microbiology, and laboratory animal breeding. During these years, Dr. Pourtaghva also played a significant role in controlling infectious diseases in the country (Figure 3).

 Dr. Pourtaghva’s work records include being transferred to the regional health and welfare organization of Tehran province in 1980. He served as the head of Najmiyeh Hospital and the head of the Epidemiology and Statistics Department of the Centre for Communicable Disease Control of the Ministry of Health from 1982 to 1985. Even after retirement, he continued to assist the Ministry of Health as a consultant for combating communicable diseases and a member of the national committee for the fight against infectious diseases.

 After his retirement, Dr. Pourtaghva continued to serve in the treatment of infectious diseases in different hospitals in Tehran. Additionally, he provided important services to support the health of warriors during the imposed war and participated in control programs for Leishmaniasis and scorpion stings on southern fronts. He also collaborated with notable figures such as Dr. Mansour Shamsa, Dr. Ahmed Jalili, and Dr. Delaram Arin with Daneshmand journal.

 Three important parts of his activities at Pasteur Hospital, the production of the smallpox vaccine and the changing of the rabies vaccination procedure in Iran have been reviewed in the following sections.

## Improvement of Pasteur Hospital

 During the presidency of Dr. Mehdi Ghodssi at the Pasteur Institute of Iran in 1965, French architect Mr. Andre Julien constructed Pasteur Hospital on the northwest side of the institute. Originally intended as a sanatorium for those seeking anti-rabies treatment, it could accommodate up to fifty patients. However, as the anti-rabies treatment network expanded across the country with the help of the Pasteur Institute of Iran, the hospital’s capacity increased, and it became the foundation of an infectious disease research hospital.^5^

 Dr. Mostafa Pourtaghva served as the head of Pasteur Hospital for a decade (from 1972 to 1981) and made significant contributions to expanding the institute’s research efforts in solving clinical problems related to infectious diseases such as tuberculosis, plague, cholera, infant tetanus, and rabies.

## Production of the Smallpox Vaccine

 The Pasteur Institute of Iran was established with the primary goal of providing vaccines and promoting vaccination. Dr. Joseph Mesnard, the first director of the institute, brought the original material for the smallpox vaccine from Paris to Iran in April 1922. After the production of this vaccine at the Pasteur Institute of Iran, Mesnard made it available to the public. In June 1929, a mandatory vaccination bill was passed, making abstinence from smallpox vaccination punishable. In June 1941, a plan to prevent communicable diseases was approved, mandating smallpox vaccination at 2 months, 7 years, 13 years, and 21 years of age. By 1936, the institute was producing 4‒5 million doses of smallpox vaccine annually, and by 1947, the production had increased to 50 million doses per year.^6^

 Dr. Pourtaghva served as the head of the smallpox vaccine production laboratory at the Pasteur Institute of Iran from 1965 to 1971. During this time, the institute expanded its program to eradicate smallpox worldwide and exported its vaccine to countries such as Turkey, Pakistan, Saudi Arabia, and Ethiopia. In 1970 alone, 35 million doses of the vaccine were exported, generating three times the institute’s annual budget. The vaccine produced by the Pasteur Institute of Iran, under the guidance of the Ministry of Health’s smallpox office, helped reduce the number of smallpox patients and deaths each year. Iran’s fight against smallpox began in 1955, and by 1964, the disease had been eradicated from the country. Smallpox vaccine production at the Pasteur Institute of Iran continued until 1977, with most of the vaccine being exported abroad.^6,7^

## Changing the Rabies Vaccination Procedure in Iran

 At the Pasteur Institute of Iran, Dr. Mahmoud Bahmanyar and Dr. Marcel Baltazard reported on the success of serum therapy in treating rabies in individuals with deep wounds.^8^ However, deaths from rabies continued to occur despite the combined use of vaccines and serums. The discovery of a cellular anti-rabies vaccine at the Mérieux Institute in France led to its purchase and use throughout Iran. The cellular vaccine was easy to administer, with a short course of less than ten days and minimal side effects.

 The sales representative of Mérieux Institute products in Tehran imported the cellular rabies vaccine and provided it to the Pasteur Institute of Iran at a cheap price, as the Pasteur Institute could officially assess and announce the Mérieux Institute’s cell vaccine as soon as possible. Dr. Mahmoud Bahmanyar and Dr. Mostafa Pourtaghva evaluated the vaccine on fifty individuals and confirmed its safety and efficacy, leading to its replacement with the old nervous tissue vaccine in Iran.^9^

 In line with his activities in the fight against rabies, Dr. Pourtaghva has also participated in a study that evaluated rabies vaccines (Human diploid cell vaccines) for ten years.^10^

## Other Scientific Activities

 More than 30 papers have been published by Dr. Mostafa Pourtaghva in medical and scientific journals in Iran and around the world.

 Dr. Pourtaghva researched various diseases, including hepatitis B and C, leprosy, and hemorrhagic fevers, during his studies in Senegal. One significant study he participated in revealed that mosquitoes and bed bugs, specifically *Cimex hemipterus*, have the potential to carry the hepatitis B virus.^11^

 In addition, he conducted studies on diseases prevalent in his hometown in northern Iran. In 1975, he investigated the presence of *Burkholderia pseudomallei* in soil samples from rice fields and found that this highly pathogenic bacteria was circulating in the area.^12^ He also identified *Pseudomonas pseudomallei* as the possible cause of a skin disease observed in people who worked in rice fields in Gilan and Mazandaran regions in 1974 and 1975.^13^ Dr. Pourtaghva further conducted extensive research on botulism, which is mostly transmitted from fish and commonly reported in Gilan province.^14,15^ Additionally, he performed studies on fasciolosis, an endemic disease of Iran that has the highest number of cases in Gilan province, to better control its spread in the northern region.^16^

**Figure 4:**
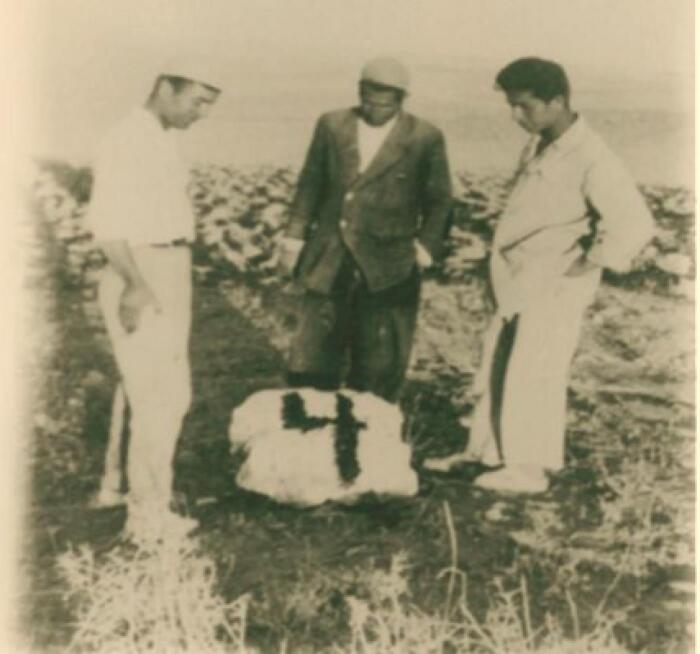
From the Right: Dr. Mostafa Pourtaghva, Mohammad Hanifi, and Dr. Houshang Majid Teymouri, a Numbered Stone in the Plague-prone Area of Agh Bolagh Morshed in Hamedan, to Mark the Locations Where Rodents Infected With the Disease Have Been Identified (1949)

**Figure 5:**
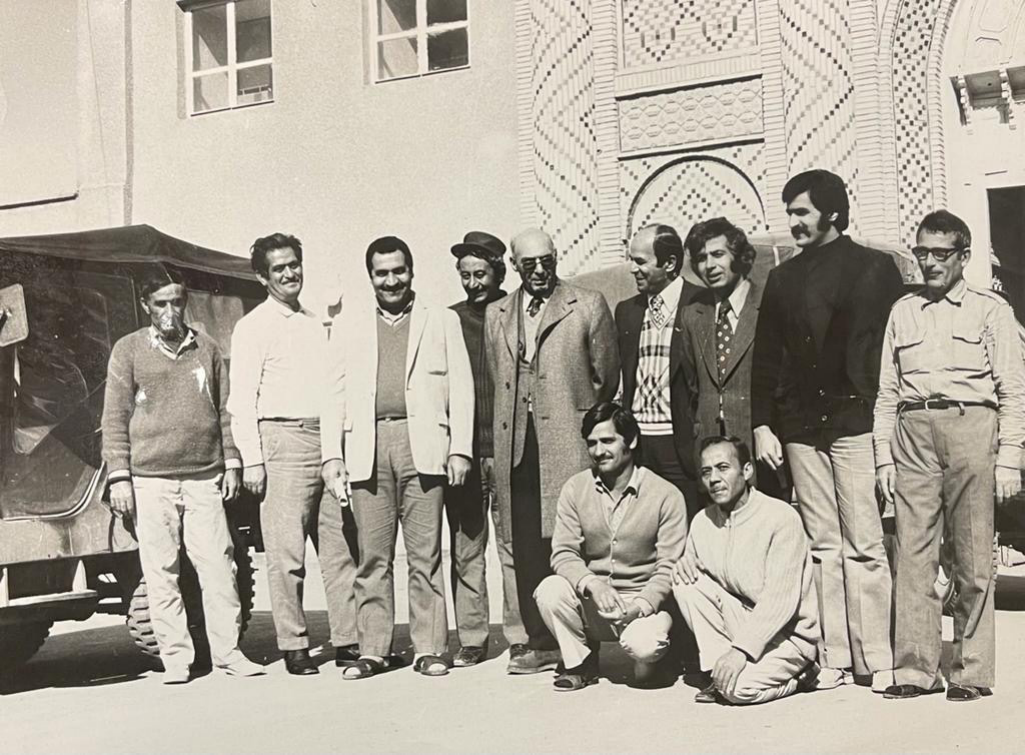
Standing From the Right: Asadollah Barandak, Abbas Azarnia (Babrzadeh), Dr. Mostafa Pourtaghva, Dr. Houshang Majid Teimuri, Dr. Sabar Mirza Farman Farmaian, Hadi Dolatabadi, Mirazim Ghasemi, Vahab Hazrati, and Valeria Sarmiyaho; Seated From the Right: Kazem Arghandeh, Hashem Baheri, Research Center for Emerging and Re-emerging Infectious Diseases of Pasteur Institute of Iran, Akanlu, KabudarAhang, Hamedan, 1958.

**Figure 6:**
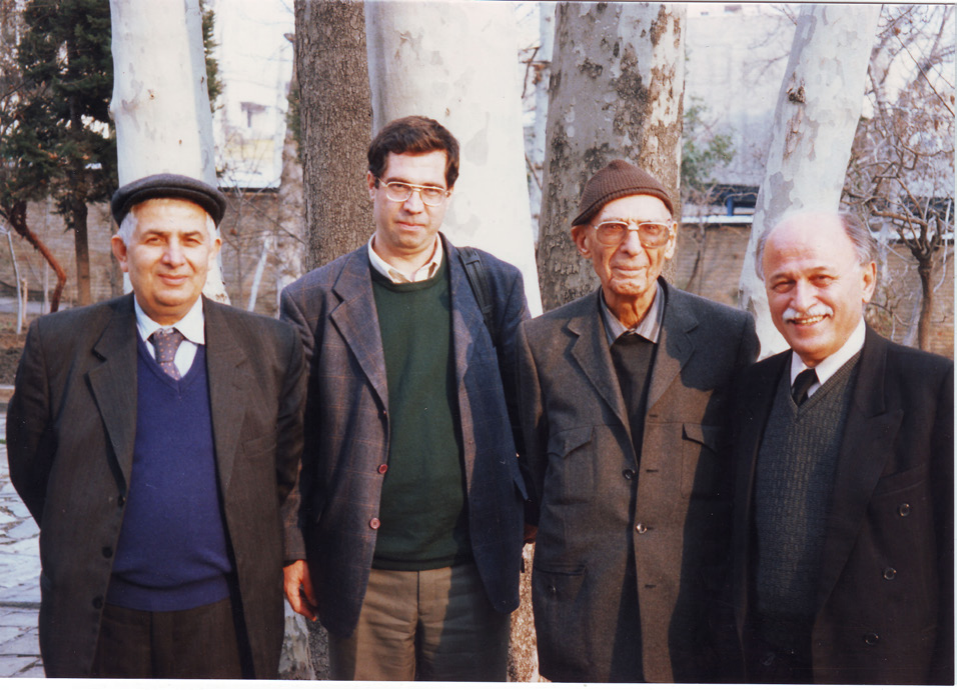
From the Right: Dr. Behrouz Broumand, Dr. Sabar Mirza Farman Farmaian, Mr. Ali Dehbashi, and Dr. Mostafa Pourtaghva at Dr. Sabar Mirza Farman Farmaian’s House, December 2000.^19^

 Dr. Pourtaghva also conducted extensive research on plague outbreaks in Iran, working with the Pasteur Institute of Iran groups under the supervision of Dr. Marcel Baltazard, alongside Dr. Younes Karimi, Dr. Mahmoud Bahmanyar, Dr. Mansour Shamsa, Dr. Biuk Seyyedian, and others.^17^ Their research identified rodents as essential in maintaining and transmitting the *Yersinia pestis*, controlling plague outbreaks in the west and northwest of the country, and identifying rodents sensitive and resistant to plague^18^ (Figures 4 to 6).

 Additionally, he has researched rabies^8,20^ and brucellosis.^21^ Furthermore, he contributed to the development of techniques for diagnosing non-dysenteric amoebic colitis^22^ and distomatosis in Iran.^23^ In 1976, he reported the first case of human pulmonary melioidosis in Iran.^24^

 In December 2021, one of the streets of Akanlu village, where the research center for emerging and reemerging infectious diseases of the Pasteur Institute of Iran is located, was named after Dr. Mostafa Pourtaghva for his services in controlling the plague and other infectious diseases.

 In 1992, the city of Amol in Mazandaran Province hosted the first inaugural National Congress of Zoonotic Diseases in Iran. The event was organized by Dr. Mostafa Pourtaghva Shahrestani and other physicians and veterinarians from the Pasteur Institute of Iran, the University of Tehran’s Faculty of Veterinary Medicine, and other universities who shared an interest in studying the epidemiology of zoonotic diseases in Iran.

## Character

**Figure 7:**
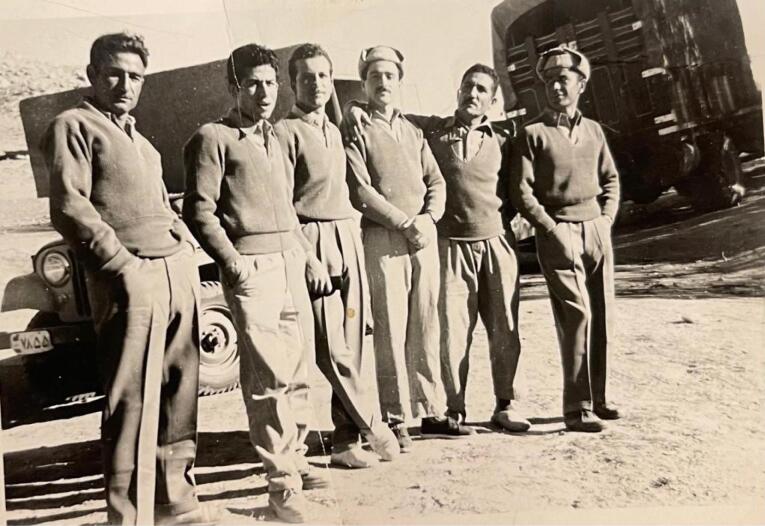
From the Right: Dr. Houshang Majd Teimuri, Salman Khan (Driver and Cook), Abbas Azarnia, Hamed Salarkia, Dr. Mostafa Pourtaghva, and Vahab Hazrati, Research Center for Emerging and Re-emerging Infectious Diseases of the Pasteur Institute of Iran, Akanlu, KabudarAhang, Hamedan, 1960.

 Dr. Pourtaghva considers it the honor of his working career to be a student of Dr. Marcel Baltazard, Dr. Mansour Shamsa, and Dr. Sabar Mirza Farman Farmaian (Figure 7).

 Dr. Mehdi Ghodssi, in his book, has described Dr. Pourtaghva as a good-natured doctor, and most of the employees of the Pasteur Institute of Iran are indebted to him for his unrelenting treatments.^9^

 Despite his many accomplishments, Dr. Pourtaghva is described as a humble and kind doctor who loves people and considers teamwork to be crucial in research. He believes that, as a Pasteurian, he has to research and serve humanity.

## Conclusion

 Dr. Mostafa Pourtaghva Shahrestani has been a prominent figure in public health in Iran, with an impressive track record of valuable contributions to Iran throughout his lifetime. His dedication and hard work serve as an exemplary model for the youth of the region.
